# Medical oncology outpatients’ preferences and experiences with advanced care planning: a cross-sectional study

**DOI:** 10.1186/s12885-019-5272-6

**Published:** 2019-01-14

**Authors:** Amy Waller, Heidi Turon, Jamie Bryant, Alison Zucca, Tiffany-Jane Evans, Rob Sanson-Fisher

**Affiliations:** 1 Health Behaviour Research Collaborative, School of Medicine and Public Health, Faculty of Health and Medicine, University of Newcastle, Callaghan, NSW Australia; 2 Priority Research Centre for Health Behaviour, University of Newcastle, Callaghan, NSW Australia; 3 Hunter Medical Research Institute, New Lambton Heights, NSW Australia; 4 Clinical Research Design and Statistics Support Unit, Hunter Medical Research Institute, New Lambton Heights, NSW Australia

## Abstract

**Background:**

Medical oncology outpatients are a group for whom advance care planning (ACP) activities are particularly relevant. Patient views can help prioritise areas for improving end of life communication. The study aimed to determine in a sample of medical oncology outpatients: (1) the perceived importance of participating in ACP activities; (2) the proportion of patients who have ever participated in ACP activities; and (3) the proportion of patients who had not yet participated in ACP activities who were willing to do so in next month.

**Methods:**

Adult medical oncology outpatients in two Australian cancer treatment centres were consecutively approached to complete a pen-and-paper survey. Items explored perceived importance, previous participation, and willingness to participate across key ACP activities including: discussing wishes with their family or doctor; recording wishes in a written document; appointing a substitute decision maker (SDM); and discussing life-expectancy.

**Results:**

185 participants completed the survey (51% consent rate). Most patients agreed it was important to: discuss end of life wishes with family (85%) and doctors (70%) and formally record wishes (73%). Few had discussed end of life wishes with a doctor (11%), recorded their wishes (15%); chosen a SDM (28%); discussed life expectancy (30%); or discussed end of life wishes with family (30%). Among those who had not participated in ACP, most were willing to discuss life expectancy (66%); discuss end of life wishes with family (57%) and a doctor (55%); and formally record wishes (56%) in the next month. Fewer wanted to appoint a SDM (40%).

**Conclusion:**

Although medical oncology outpatients perceive ACP activities are important, rates of uptake are relatively low. The willingness of many patients to engage in ACP activities suggests a gap in current ACP practice. Efforts should focus on ensuring patients and families have clarity about the legal and other ramifications of ACP activities, and better education and training of health care providers in initiating conversations about end of life issues.

## Background

Despite advances in treatments, cancer remains a leading cause of death worldwide [1]. High quality cancer care has been defined by the Institute of Medicine (IOM) as care that is safe, effective, patient-centered, timely, efficient, and equitable [2]. Quality indicators of end-of-life care have been developed globally, however there is wide variation across care domains and service providers [3–5]. There has been increasing recognition over the past decade that many cancer patients receive aggressive medical care at the end of life [6], including receipt of cancer-directed procedures and therapies; emergency room and intensive care admissions; and in-hospital deaths [3, 4]. Between 5 and 55% of patients with advanced cancer receive chemotherapy in the last month of life [7]. A US study of 28,731 patients aged < 65 years found 71–76% of patients with varied cancer types received aggressive care in the last 30 days of life, including 30–35% of patients who died in the hospital [8]. It is estimated that between 33 and 38% of inpatients will receive futile treatment [9]. In addition to clinical criteria, inappropriate hospital admissions at the end of life have been attributed to poor availability of alternative care options, failure of preventive actions by healthcare providers; family requests; or too late an admission to be of benefit [10]. While aggressive medical care often results in disproportionately greater health expenditure, it may not correspond to increases in length or quality of life for patients. An Australian study found that overall health care costs were significantly higher among those who died from cancer than those dying from other causes, with 40% of costs expended in the last month of life [11].

ACP is an “ongoing process that supports adults at any age or stage of health in understanding and sharing their personal values, life goals, and preferences regarding future medical care” [12]. It can include: making a written document (such as an advance care directive) to express values and instructions about health matters and/or the appointment of substitute decision makers (SDM) to make health and personal decisions during periods of incapacity [12, 13]. The benefits of ACP are well established. For example, engaging in ACP has been shown to reduce non-essential transfers to hospital and decrease life-sustaining treatment at the end of life [14], improve family satisfaction with end-of-life care [15, 16], and increase use of hospice and palliative care [16]. ACP has been shown to increase the concordance between preferred care and the care actually delivered. Despite its potential benefits however, some evidence suggests that ACP is not systematically implemented in cancer care. The reported proportion of advanced cancer patients who engage in end-of-life discussions varies, with some reporting fewer than 40% [16, 17] and others as high as 73% [18, 19]. Rates of documentation of ACP with an oncologist have been as low as 10%; with rates of documentation increasing to between 30 and 40% in intervention studies [20].

Extensive research has examined the factors influencing uptake and the utility of ACP for individuals with cancer. A recent systematic review examined patient, caregiver and healthcare provider experiences and perceptions of ACP in cancer care, and concluded that uptake of ACP is a function of a range of complex relational, emotional and social factors [21]. Studies examining barriers to ACP report that unrealistic patient or family expectations of prognosis and poor understanding of care outcomes can hinder timely end-of-life conversations [22]. Limited provider time, skill and confidence in implementing ACP are also recognised as important barriers [23]. However, our knowledge of the extent to which ACP activities are valued by cancer patients, particularly in the Australian context, is uncertain [21]. Prospective surveys that assess participation in ACP activities at a particular time point should be interpreted in the context of a patients’ readiness to engage in activities [24]. Understanding patient willingness to participate in ACP activities can provide greater clarity about whether rates of uptake represent a gap in care or are a result of patient preference.

Therefore, this study examined the perceived importance, rate of uptake, and preferences for participation in ACP activities in a sample of medical oncology outpatients attending two Australian cancer treatment centres. Specifically, the aims of this study were to determine in a sample of medical oncology outpatients, the:

1. perceived importance of participating in key advance care planning (ACP) activities;
2. proportion of patients who had already participated in ACP activities; and
3. proportion of those who had not already participated in ACP activities who were willing to so in the next month.

## Methods

### Design

A descriptive cross-sectional survey of medical oncology outpatients recruited from two Australian tertiary cancer treatment centres located in metropolitan areas.

### Participants

Eligible patients had a confirmed cancer diagnosis, were attending the clinic for their second or subsequent appointment, were at least 18 years old, were able to read and understand English, and were deemed by clinical staff to be physically and mentally able to give informed consent and to complete the survey.

### Procedure

A description of the procedure for the baseline survey has been described in detail previously [25]. Briefly, potentially eligible patients were identified by clinic staff from clinic lists. Eligible consecutive patients were approached by research staff while in the clinic waiting room and invited to participate and written consent obtained. Consenting patients completed a paper and pen baseline survey assessing demographic and clinical characteristics while waiting for their appointment. Alternatively, participants could complete the survey at home and post it back to the research team in a provided reply paid envelope. The baseline survey included questions pertaining to participant demographics, cancer and treatment information, anxiety, depression and unmet needs. A second survey was mailed to participants 4 weeks later. In addition to the ACP items described below (see outcome measures), the second survey included items developed specifically for the study assessing: access to and use of the internet, acceptability of researchers accessing personal and health information including Medicare and the Pharmaceutical Benefits Scheme, and preferred methods for communicating treatment information, such as risks of side effects and survival. Non-responders to both surveys received reminder surveys after 2 weeks and a further 4 weeks. Ethics approval was obtained from University of Newcastle (H-2010-1324) and the participating centres.

### Outcome measures

Given the potentially sensitive nature of the items, patients could opt out of completing the questions pertaining to ACP. For those participants who chose to complete the ACP questions, the following topics were explored:

#### Importance of participating in ACP activities

Patients were asked to respond to the following statement: “In case you are unable to make decisions later, do you think it is important that you”: (1) “*Talk with your family about the type of end of life care you would want to receive*”; (2) “*Talk with your doctor about the type of end of life you would want to receive*”; (3) “*Record the type of care you would want to receive in a written document (i.e. advance care directive)*”; and (4) “*Formally choose someone to make decisions about your care on your behalf (i.e. a surrogate decision maker)*”. For each item, respondents selected either: strongly agree, somewhat agree, somewhat disagree or strongly disagree*.*

#### Self-reported participation in ACP activities

Patients were asked whether they had already: (1) “*Talked with your family about the type of end of life care you would want to receive*”; (2) “*Talked with your doctor about the type of end of life you would want to receive*”; (3) “*Recorded the type of care you would want to receive in a written document (i.e. advance directive)*”; and (4) “*Formally chosen someone to make decisions about your care on your behalf (i.e. a surrogate decision maker)*”; and (5) “*Discussed how cancer may affect the length of your life (your life expectancy) with your doctor*”. Response options for each item were ‘yes’, ‘no’ or ‘unsure’.

#### Willingness to participate in ACP activities

Patients were asked to respond to the following statement: “If you were given the opportunity in the next month, would you choose to”: (1) *“Discuss your preferences for end of life care with your family”*; (2) “*Discuss your preferences for end of life care with your doctor”*; (3) *“Record the type of care you would want to receive in a written document (i.e. complete an advance directive)”*; (4) *“Formally choose someone to make decisions on your behalf (your surrogate decision maker)”*; and (5) *“Discuss how cancer may affect the length of your life (your life expectancy) with your doctor”*. Response options were ‘yes’, ‘no’ or ‘unsure’.

#### Sociodemographic and disease variables

Participants also reported a number of sociodemographic and disease variables including age, gender, education, country of birth, marital status, type of cancer, time since diagnosis, stage at diagnosis, remission status and treatments received. Variables are summarised as frequencies, and percentages of non-missing responses.

### Statistical analyses

Statistical analyses were programmed using SAS v9.4 (SAS Institute, Cary, North Carolina, USA). A composite ACP score was created by summing the number of ‘yes’ responses for these four items (i.e. discussions with doctor, discussion with support person, written end of life wishes and appointed surrogate decision maker). Scores ranged from 0 (none) to 4 (all). Missing values assumed a value of 0. A sample size of 185 allowed estimation of population proportions within a 7% margin of error.

## Results

### Sample

A total of 608 patients were screened for eligibility at the two participating treatment centres. One hundred and seventeen were ineligible for the following reasons; no confirmed cancer diagnosis (*n* = 23), non-English speaking (*n* = 36), too sick (*n* = 25), first visit (*n* = 21), other reasons (*n* = 19). Some patients were ineligible for multiple reasons. Of the remaining 491 patients, 361 consented (consent rate = 74%). Of these, 300 (83%) completed and returned the baseline survey. Of the 217 patients who completed the follow-up survey, 185 (88%) opted to complete the end of life questions (see Table 1). The mean age of participants was 65 years (SD = 11.54).

**Table 1:** Characteristics of study sample (*n* = 185)

Variable	Category	Number	Percent
Gender	Male	35	19
	Female	146	81
Marital status	Single, divorced, separated or widowed	61	34
	Married or in a relationship	119	64
Highest level of education	Primary school (year 6)	7	4
	High school	86	48
	Trade or University	80	44
	Other	7	4
Living arrangements	Spouse/partner/children	127	71
	On my own	35	19
	Other family members	14	7.8
	Other	4	2.2
Country of birth	Australia	128	71
	Other	52	29
Months since diagnosis	Less than 6 months	45	25
	7–12 months	34	19
	13–24 months	29	16
	More than 24 months	73	40
Treatment received to date^a^	Surgery	133	73
	Chemotherapy	148	82
	Radiotherapy	93	52
Cancer type	Breast	88	49
	Colorectal	14	7.8
	Haematology	13	7.3
	Melanoma	12	6.7
	Lung	8	4.5
	Prostate	4	3
	Other	40	22
Stage at diagnosis	Early	105	60
	Progressed / advanced	58	33
	Don’t know	10	5.7
Remission status	Not in remission	82	45
	In remission	50	28
	Don’t know	49	27

^a^Patients could indicate receiving multiple treatment types

### Do patients think it is important to communicate and document end of life wishes?

The perceptions of patients about each ACP activity are outlined in Table 2. The majority of patients strongly agreed it was important to discuss end of life wishes with their family (85%). Most also strongly agreed it was important to discuss end of life wishes with their doctor (70%) and to record wishes in a written document (73%). Fewer patients strongly agreed that it was important to formally choose a surrogate decision maker (53%).

**Table 2:** Patients views about the importance of each of the ACP activities

ACP activities	Strongly agree N (%)	Somewhat agree N (%)	Somewhat disagree N (%)	Strongly disagree N (%)
Talk to family about end-of-life wishes	152 (85%)	25 (14%)	0 (0%)	2 (1.1%)
Talk to doctor about end-of-life wishes	124 (70%)	45 (25%)	5 (2.8%)	4 (2.2%)
Record wishes in a written document	132 (73%)	37 (20%)	10 (5.5%)	3 (1.6%)
Choose a surrogate decision-maker	95 (53%)	52 (29%)	20 (11%)	12 (6.7%)

### Have patients participated in ACP activities?

Participation in each of the examined ACP activities is provided in Table 3. Just over a third of patients had already discussed the type of care they would want to receive with their family (*n* = 66, 36%); however, only 11% had discussed this with their doctor. Almost a third (30%) had discussed their life expectancy with their doctor. Few indicated they had recorded their wishes in a written document (15%), while 28% had appointed a surrogate decision maker. Overall, 38% had not participated in any ACP activity; while 5.5% had participated in all ACP activities. Compared to those with early disease, a significantly higher proportion of participants diagnosed with advanced disease had appointed a SDM (25% vs 34%, *p* = 0.0226). There were no differences in the proportion of early vs advanced patients who had: completion of ADs (16% both sub-groups), end of life discussions with family (36% vs 41%), end of life discussions with doctor (12% vs 13%) or life expectancy discussions (35% vs 30%).

**Table 3:** Participation in ACP activities

ACP activities: Have already	Yes N (%)	No N (%)	Unsure N (%)
Talked to family about the type of end of life care s/he would want to receive	66 (35.6)	107 (57.8)	4 (2.2)
Talked to doctor about the type of end of life care s/he would want to receive	20 (10.8)	154 (83.2)	2 (1.1)
Recorded the type of care s/he would want to receive in a written document	28 (15.1)	149 (80.5)	2 (1.1)
Formally chose someone to make decisions about care	52 (28.1)	121 (65.4)	4 (2.2)
Discussed life expectancy with his/her doctor	55 (29.7)	119 (64.3)	3 (1.7)

**Table 4:** Willingness of patients who had not participated in ACP activities already to do so in the next month

ACP activities	Yes N (%)	No N (%)	Unsure N (%)	Total n
Talked to family about the type of end of life care s/he would want to receive	61 (55%)	21 (25%)	28 (19%)	111
Talked to doctor about the type of end of life care s/he would want to receive	89 (57%)	29 (19%)	37 (24%)	156
Recorded the type of care s/he would want to receive in a written document	84 (56%)	25 (17%)	42 (28%)	151
Formally chose someone to make decisions about care on his/her behalf	50 (40%)	34 (27%)	38 (30%)	125
Discussed life expectancy with his/her doctor	81 (66%)	23 (19%)	16 (13%)	122

### Are patients who have not participated in ACP willing to do so in next month?

As shown in Table 4, among those who had not already participated in each ACP activity, 66% wanted to talk to their doctor about their life expectancy in the next month. Many also wanted to talk to their family (55%) and their doctor (57%) about the type of end-of-life care they wanted. Just over half of participants wanted to formally record their wishes in a written document (56%), while 40% wanted to appoint someone as their substitute decision-maker in the next month.

## Discussion

The value of ACP as a key component of optimal care is increasingly acknowledged by professional oncology organisations and in practice and policy documents [18]. This heterogeneous sample of medical oncology outpatients, of mixed cancer types, at various stages in the cancer care pathway, appeared to share this view. Discussions with families about end-of-life care wishes was particularly valued by patients. This is consistent with previous studies in which patients who choose to engage in end-of-life conversations typically prefer their family be involved [26]. The majority of patients also acknowledged the importance of having end-of-life conversations with doctors. Health professional involvement helps to ensure that patients and their families hold accurate views about end-of-life issues such as prognosis and treatment intent [27, 28]; and that decisions are appropriate to patient circumstances. Fewer patients viewed the appointment of substitute decision makers as important when compared to other ACP activities. It may be that patients believe family members already know and will enact their wishes without the need for formal appointments; or wish to avoid burdening family with this role [21].

The positive views held by patients did not consistently translate into action, with only one third of respondents having actually discussed their wishes with their family. Previous studies suggest that patients may avoid discussions if they have concerns that confronting end-of-life issues may have negative impact on family members [21, 29]. Very few had already spoken to their doctor about the type of end-of-life care they would like to receive, and less than one third had discussed life expectancy with their doctor. This low uptake is consistent with previous studies in similar populations [16–18]. While not here, low recall by patients that discussions have occurred; or provider- or other system-related barriers (e.g. lack of provider skills, fear of depriving patient of hope) have been reported to contribute to low reported rates of discussion between patients and health care providers [21, 23].

Despite recommendations that more formal ACP activities be a routine part of care [30], very few patients had recorded their wishes in a written document, such as an advance care directive (ACD). Increasingly, the field is moving toward implementing ACP as a process that encompasses ongoing discussions and the appointment of substitute decision-makers; rather than relying solely on the completion of ACDs [31]. The low rate of written documentation reported by respondents in this study may therefore reflect this change in focus. Previous studies also show that some patients are unaware of the availability of legal written documents such as ACDs or their function [32]. Some express concerns about not being able to change written documents [33], preferring instead that decisions are made at the time by their health care team and/or family members. Family members may also play a role in a person’s decision to complete ACDs [34]. Only one third of respondents reported having already appointed a SDM, perceiving this as less important when compared to other ACP activities. This is despite SDM appointments having been identified as one of the key outcomes of successful ACP [13]. There is a need to ensure patients and families are aware of the purpose and potential benefits of appointing substitute decision makers and formally documenting wishes.

Despite the positive attitudes held by the majority of patients towards the range of ACP activities described, the lack of participation in ACP activities appeared concordant with the current preferences of some patients. A perceived lack of importance may have contributed to unwillingness to participate, particularly for activities such as appointing a SDM. Given the majority of patients perceived ACP activities as important in this study, it may be that patients instead did not feel that these activities were relevant to them yet. As many patients perceived their cancer to be curable or in remission, the one-month timeframe may also have been a factor. That many patients, including those who self-reported having advanced disease, had not already participated in the ACP activities did express a desire to do so suggests there is a gap between what patients want and what they are actually receiving in relation to informal and formal end-of-life communication.

### Clinical implications

ACP is comprised of multiple behaviours, such as identifying one’s values, choosing a SDM, and discussing values with SDMs and clinicians, as well as recording wishes in written documents [13]. Widespread uptake in ACP in health systems has been achieved through a range of initiatives, including public awareness campaigns that promote ACP in the general community and approaches incorporating provider education and training, mechanisms to identify and trigger early conversations, and continuous monitoring [18, 35–37]. Our findings reveal that many cancer patients are willing to engage in ACP activities if given the opportunity, even though they may not be facing the end of their life. Better education and training of clinicians is needed to support the identification of those patients who are willing to participate in ACP activities. Patients should be given opportunities to explicitly communicate end-of-life wishes in writing or verbally to nominated SDMs and health care providers, as these individuals may still default to providing all care possible when wishes have not been communicated clearly [18]. With patient consent, substitute decision makers should be involved in each step of the ACP process, including ongoing clinician-patient discussions of life expectancy, goals of care, and preferences for end-of-life treatments [18]. Willingness to participate in ACP activities should be explored routinely, as preferences may change as health or personal circumstances alter.

### Limitations

The findings of this study should be considered in light of several limitations. Firstly, like many studies in this field, the sample of cancer patients surveyed was heterogeneous and at different stages of their cancer journey, and may have included those who perceived end of life conversations not relevant to their specific circumstance. This may have influenced our findings related to willingness to participate in ACP in the next month. The low response rates may be due to some patients taking surveys home to complete, or the nature of the topic. We also did not ask about current stage of disease, which is a limitation of the study. Our previous study in an oncology population found that those who perceived their cancer as incurable were more likely to have participated in ACP [38]. Perceived importance of prognosis disclosure was not examined. However, previous studies in the literature indicate that the majority of patients perceive this to be an important part of their cancer care [39–41]. Additionally, restricting the question to willingness to participate in ACP in the next month- rather than a longer timeframe- may have also reduced the number of participants who were willing to engage. Due to sample size and design considerations, association between outcomes and socio-demographic and disease variables were not examined. Associations by cancer type and stage may provide important information of use to clinicians to guide timing of discussions about ACP, and should be explored.

## Conclusion

Advance care planning aims to support patients and SDMs to participate with clinicians in making the best possible in-the-moment medical decisions. While medical oncology outpatients hold positive attitudes toward key ACP activities, these attitudes do not consistently translate to ACP uptake. This is despite many patients who have not participated in these activities expressing a willingness to do so. Efforts to promote the adoption of ACP in oncology should focus on ensuring patients and families have clarity about the legal and other ramifications of ACP activities, particularly the importance of designating SDMs; and better education and training of health care providers in initiating conversations about end-of-life issues.

## Abbreviations

ACP: Advance care planning
AD: Advance directive
IOM: Institute of Medicine
SDM: Substitute decision-maker
UK: United Kingdom
USA: United States of America
